# A Case of Highly Myopic Strabismus That Showed Temporary Large Exotropia After the Yokoyama Procedure

**DOI:** 10.7759/cureus.88932

**Published:** 2025-07-28

**Authors:** Anzu Hirota, Toshiaki Goseki, Keiko Kunimi, Masumi Kimijima, Yoshiaki Ichibe

**Affiliations:** 1 Department of Ophthalmology, Kanagawa Dental University Yokohama Clinic, Yokohama, JPN; 2 Department of Ophthalmology, International University of Health and Welfare Atami Hospital, Atami, JPN; 3 Department of Ophthalmology, Kitasato University, Sagamihara, JPN

**Keywords:** esotropia, exotropia, highly myopic strabismus, postoperative management, yokoyama procedure

## Abstract

Highly myopic strabismus is a progressive form of esotropia and hypotropia. It occurs when the globe axis is elongated due to high myopia, causing the posterior portion of the globe to dislocate between the superior rectus and lateral rectus muscles and prolapse from the muscle cone. This condition is often accompanied by mechanical restrictions of eye movement. If it progresses, the globe may become fixed in an inferomedial position. The most common treatment is uniting the superior and lateral rectus muscle bellies using a suture posterior to the muscle insertions, also known as the Yokoyama procedure. This case report presents the case of a 59-year-old male patient who underwent the Yokoyama procedure. After surgery, the patient experienced severe inflammation and pain during eye movement; however, as the symptoms subsided, diplopia improved, and by the one-month follow-up examination, the pain had completely resolved. In addition, the patient showed a significant angle of exotropia, which spontaneously recovered to the normal position one month after the surgery, accompanied by the resolution of diplopia. When the patient underwent the Yokoyama procedure after seven years of observation following the initial examination, it was found that the patient had exotropia immediately after surgery, but the eye position improved over time, and the patient was eventually able to maintain a normal position. Postoperative magnetic resonance imaging (MRI) showed that the position of the superior rectus and lateral rectus muscles had improved, and the angle of dislocation had also improved. There have been no reports of spontaneous improvement in postoperative exotropia, as in this case, which provides new insights into the possibility of temporary changes in eye position and spontaneous improvement in the treatment of highly myopic strabismus. Additionally, it also prompts reconsideration of the importance and significance of postoperative management.

## Introduction

Strabismus can develop due to degeneration of the lateral rectus (LR)-superior rectus (SR) band, the most fragile component of the orbital pulley system, a key connective tissue structure responsible for maintaining ocular alignment [1]. When this degeneration is age-related, it may lead to sagging eye syndrome (SES), characterized by small-angle esotropia and cyclovertical strabismus [2]. In patients with high myopia, defined as a spherical equivalent of −6.00 D or greater, or an axial length ≥26 mm, the elongated posterior globe may herniate between the LR and SR muscles and extend outside the muscle cone [3-5]. This pathological condition, termed highly myopic strabismus or high myopic esotropia, is progressive and characterized by inward and downward displacement of the globe, producing esotropia with hypotropia [6,7]. As the condition progresses, it may advance to myopic strabismus fixus, a severe form of fixed esotropia in which the globe becomes entrapped in an esotropic position due to mechanical restriction [6,7]. The most common treatment for myopic strabismus fixus is the SR-LR union suture (Yokoyama procedure). The surgical procedure entails the anatomic joining of the superior rectus and external rectus muscle bellies, a process that is performed 15 mm posterior to their respective muscle attachments [6,7]. This approach is employed in the management of progressive highly myopic strabismus [8,9].

This case report details a patient who developed a significant angle of exotropia following surgery for highly myopic strabismus using the Yokoyama procedure, which returned to a normal position one month after surgery. To the best of our knowledge, there have been no reports of cases in which eye position returned to normal through follow-up observation. This case demonstrates a transient alteration in ocular alignment during the treatment of highly myopic strabismus using the Yokoyama procedure, which offers valuable insights into postoperative management.

## Case presentation

**Figure 1:**
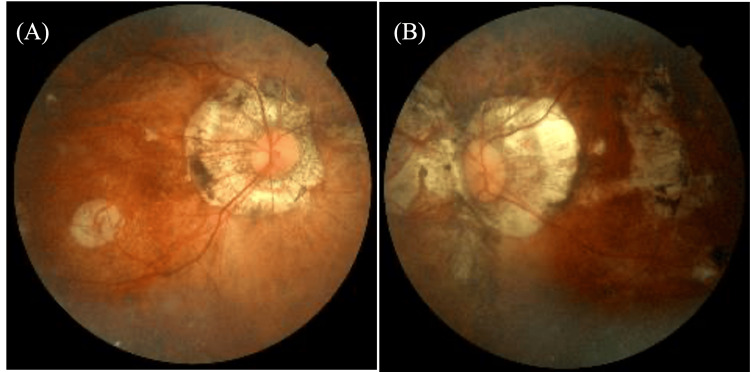
Fundus image at initial examination (A) is an image of the right eye and (B) is an image of the left eye. Retinal and choroidal atrophy can be observed in both eyes

The patient was a 59-year-old man who had been aware of distant horizontal diplopia for several years and had been wearing 2 Δ base-out prism glasses for both eyes; however, he was referred to our hospital because his symptoms had worsened. His medical history included bilateral cataract surgery at the age of 47 and glaucoma treatment with eye drops. Uncorrected visual acuity was 6/20, and corrected visual acuity was 6/5 in both eyes. Refractive examination revealed that the right eye was -2.25 -1.00 × 80 and the left eye was -2.00 -1.00 × 80. Intraocular pressure was 10.5 mmHg in the right eye and 12.6 mmHg in the left eye. No abnormalities were observed in the anterior or intermediate segment. The fundus of both eyes showed severe myopic atrophy of the choroid and retina (Figure 1).

**Figure 2:**
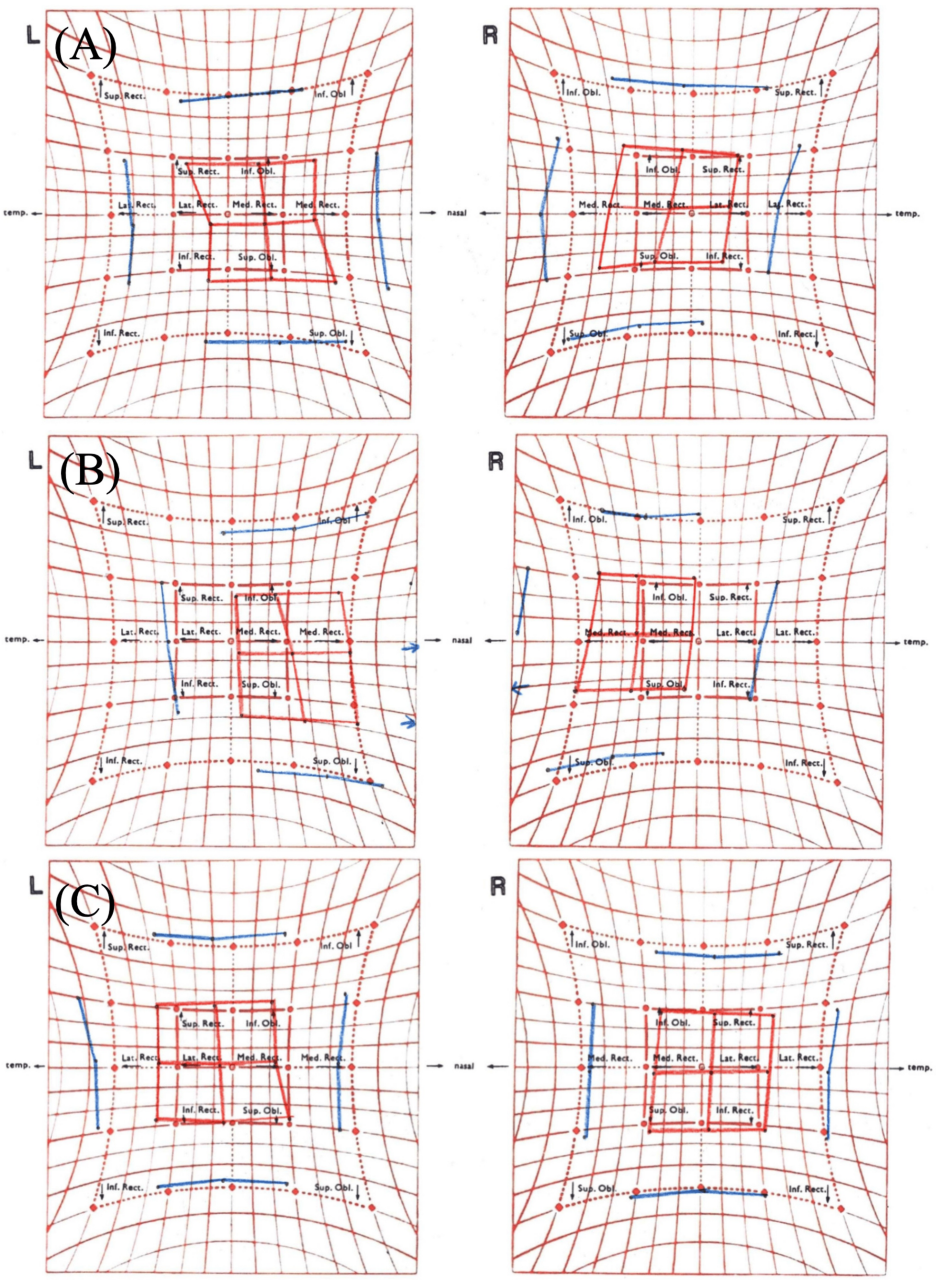
Hess chart at initial visit, before operation (seven years after initial visit) and after operation (A), (B), and (C) are images of Hess charts at the initial visit, preoperatively (seven years after the initial visit), and postoperatively, respectively. Seven years after the initial examination, the strabismus angle was observed to be worsening. Preoperative medial deviation was improved postoperatively. No obvious restriction of eye movement was observed

The axial length was long in both eyes, at 33.9 mm in the right eye and 33.0 mm in the left eye. In the alternate prism cover test (APCT), we observed 8 Δ esophoria (Ep)' 2 Δ right hyperphoria (RHp)' at near and also observed 14 Δ Ep 4 Δ RHp at distance. No obvious restriction of eye movement was observed in either eye (Figure 2A).

**Figure 3:**
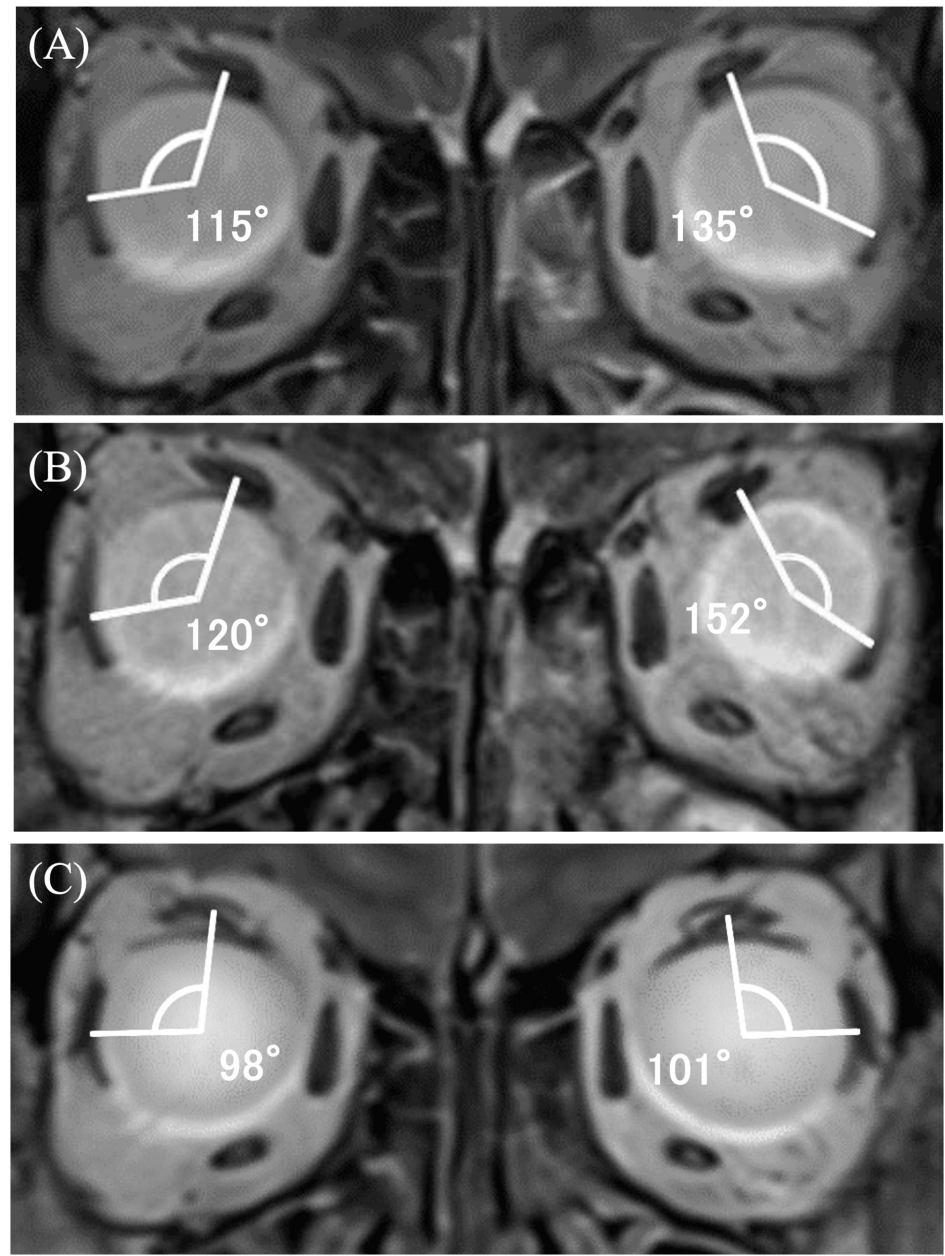
Coronal section MRI images at initial visit, before operation (seven years after initial visit), and after operation SR: superior rectus muscle; LR: lateral rectus muscle (A) is the image at the time of the initial examination, (B) is the image before surgery (seven years after the initial examination), and (C) is the image after surgery. Nasal displacement of the SR and downward displacement of the LR were observed in both eyes from the initial examination, with the angle of dislocation between the SR and the LR being 115° in the right eye and 135° in the left eye. The preoperative angle of dislocation was 120° in the right eye and 152° in the left eye, but five months after surgery, the angle of dislocation of the right and left eyes was 98° and 101°, respectively, and the angle of dislocation also showed improvement

No anomalous findings were identified on magnetic resonance imaging (MRI) of the head. On the MRI coronal section of the orbit taken 6 mm anterior to the optic nerve and the globe junction, a nasal displacement of the SR and a downward deviation of the LR were observed in both eyes. Additionally, the angle of dislocation in the orbital MRI, formed by the lines connecting the center of gravity of the SR, the center of the globe, and the LR (the angle of dislocation), was 115° for the right eye and 135° for the left eye (Figure 3a). For intermittent esotropia, the patient was prescribed prism glasses with a 5 Δ base-out for both eyes, and the outpatient follow-up was subsequently finished.

**Figure 4:**
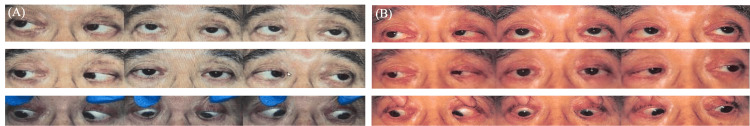
Nine gaze photos preoperatively and five months postoperatively No obvious restriction of eye movement was observed (A and B)

Seven years after the initial examination, the patient returned to our hospital with worsening subjective symptoms of diplopia, despite wearing prism glasses. The APCT demonstrated an increase in the angle of strabismus, with 50 Δ esotropia (ET) 5 Δ right hypertropia (RHT) at distance, and with 18 Δ ET' 3 Δ RHT' at near. Furthermore, the angle of dislocation in the orbital MRI also increased to 120° for the right eye and 152° for the left eye. Moreover, no obvious restriction of eye movement was observed in either eye. Consequently, the Yokoyama procedure was performed on both eyes (Figure 2B, Figure 3B, and Figure 4A).

**Figure 5:**
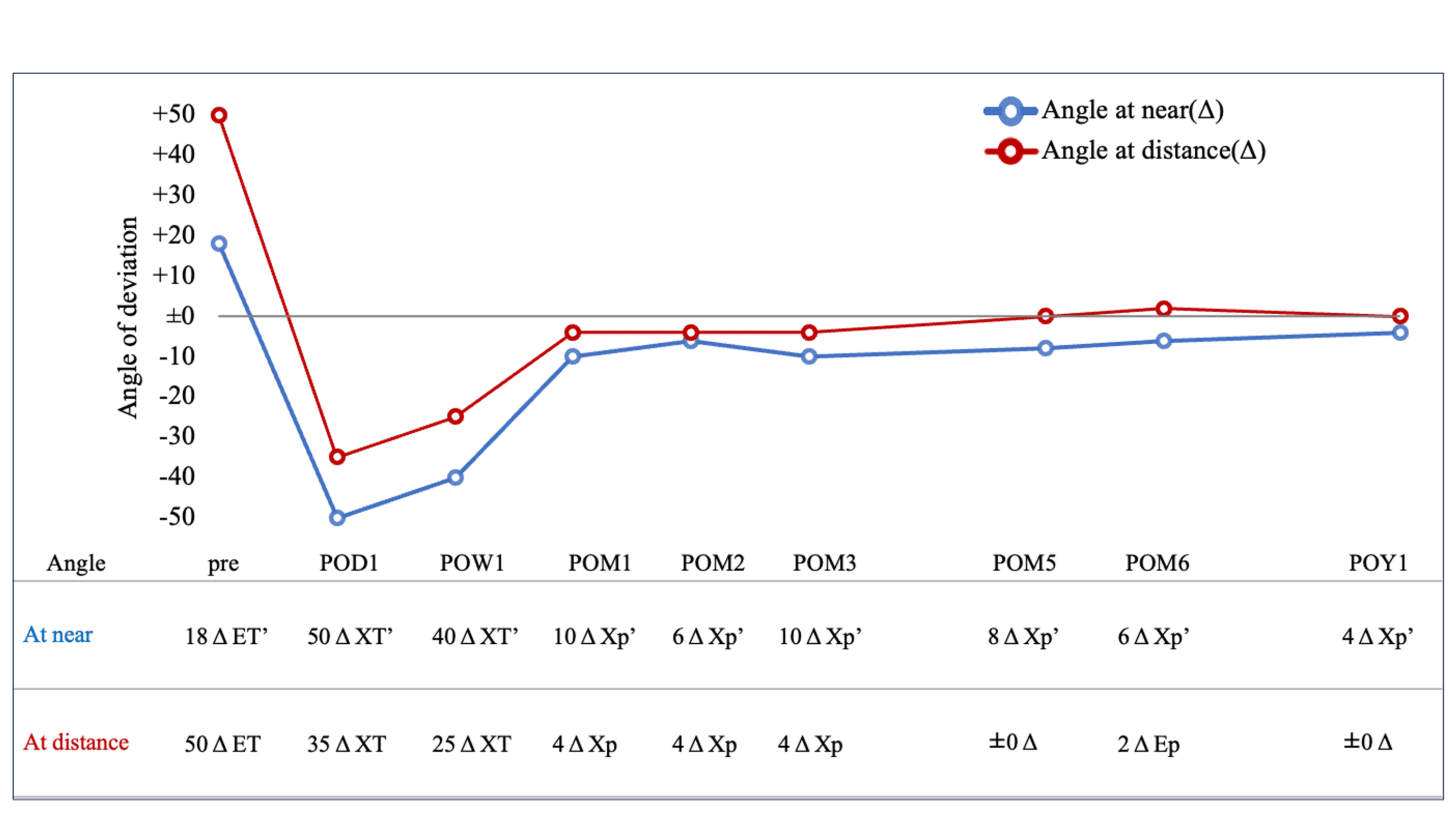
Postoperative strabismus angle changes ET: esotropia; XT: exotropia; Ep: esophoria; Xp: exophoria On the day after surgery, 50 Δ XT at near and 35 Δ XT at far were observed, which decreased to 40 Δ XT at near and 25 Δ XT at distance at one week after surgery. One month after the surgery, the values were 10 Δ Xp at near and 4 Δ Xp at distance, and the patient has maintained a normal position ever since

On the day following surgery, the APCT was 50 Δ exotropia (XT)’ at near and 35 Δ XT at distance. No discernible restriction of eye movement was observed. One week following the surgical procedure, the exotropia angle exhibited a reduction to 40 Δ XT' 3 Δ RHT' at near and 20-25 Δ XT 3 Δ RHT at distance. One month following the surgical procedure, the exotropia exhibited a notable improvement, with an angle of 10 Δ exophoria (Xp)’ at near and 4 Δ Xp’ at distance. The patient reported that the diplopia had resolved three days before the one-month postoperative examination. One year postoperatively, the patient exhibited an optimal eye position, with 4 Δ Xp’ at near and 0 Δ at distance. In the coronal section of the orbital MRI taken five months after surgery, the nasal displacement of the SR and the inferior displacement of the LR in both eyes had improved, as had the angle of dislocation, which had decreased to 98° in the right eye and 101° in the left eye (Figure 2C, Figure 3C, and Figure 4B). Figure 5 illustrates the data on the strabismus angle from the preoperative period to one year postoperatively.

## Discussion

Highly myopic strabismus is typified by a long axial length, an outward and upward deviation of the posterior part of the globe, and a dislocation angle, which worsens with age. In this case, although no obvious restriction of eye movement was observed, the long axial length, outward and upward displacement of the posterior globe, and dislocation angle were consistent with those observed in highly myopic strabismus, thereby confirming the diagnosis. The Yokoyama procedure is the most widely recommended surgical intervention. Wabbels et al. have demonstrated the efficacy of the Yokoyama procedure for the treatment of highly myopic strabismus with concomitant restriction of ocular motility [10]. In cases where the angle of dislocation is 120° or greater in both eyes, Shimizu et al. have indicated that the Yokoyama procedure is the preferred surgical approach [11]. We believe that this indication also applies to this patient. Reports indicate that the efficacy of the postoperative eye position is contingent upon the preoperative strabismus angle. However, there are also reports of cases that have resulted in overcorrection on occasion [10]. In this report, overcorrection was reported in six of 133 patients (4.5%), but the prognosis of these patients is not reported. Complications of strabismus surgery associated with unexpectedly large strabismus angles include muscle slippage, muscle loss, and pulled-in-two syndrome [12]. Nakao et al. reported a case of external strabismus caused by pulled-in-two syndrome after the Yokoyama procedure for fixed internal esotropia [13]. Following a three-month period of observation, there was no spontaneous improvement of the exotropia. Three months after the initial surgery, a second surgical procedure was performed to address anterior translation of the medial rectus muscle and posterior translation of the inferior rectus muscle.

In this case, the surgical operation was meticulously executed by a proficient surgeon, and no abnormality was detected in the attachment position of the superior rectus and lateral rectus muscles on postoperative orbital MRI. Guo et al. reported that transient strabismus and diplopia following ocular surgeries may arise not only from mechanical or surgical causes but also from decompensation of pre-existing latent strabismus due to disruption of fusional mechanisms or maladaptation of vergence control [14]. It is hypothesized that the postoperative exotropia in this case was the result of a prolonged period of divergence from the onset of the disease to the time of surgery. This prolonged period of divergence may have resulted in the temporary overactivation of divergence after surgery, leading to the manifestation of exotropia. The patient's eye position was regarded as having undergone a favorable progression as a consequence of the systematic refinement of the motion. On the other hand, Clark and Demer have shown via MRI studies that postoperative shifts in pulley positions can explain changes in eye alignment after surgery [15]. In contrast to previous reports, to the best of our knowledge, there are no other reports of spontaneous improvement of this nature. However, this particular case suggests that a follow-up period of at least one month may be feasible, provided that there are no complications, such as a loss of muscle mass in the medial rectus muscle. We believe that this case offers valuable insights that may inform the future management of highly myopic strabismus.

Limitation

This case report has several limitations. As it is a single case, the rate of spontaneous improvement of postoperative exotropia is unknown. Furthermore, the mechanism and risk factors underlying the development of large-angle exotropia cannot be confirmed based on this single case. It is essential to collect data on the efficacy and safety of the Yokoyama procedure from a larger sample size to strengthen the evidence base.

## Conclusions

In conclusion, this case report highlights a rare but important clinical course of transient large-angle exotropia following the Yokoyama procedure for highly myopic strabismus. Despite initial postoperative overcorrection, the patient's eye alignment improved spontaneously within one month, ultimately achieving stable ocular alignment without the need for reoperation. This spontaneous recovery, not previously reported in the literature, suggests that a conservative approach with careful observation may be justified in similar cases where no obvious mechanical complications are present. Furthermore, this case underscores the importance of understanding the dynamic postoperative ocular alignment process, and it raises awareness of the potential for natural rebalancing in selected patients. Future studies with larger cohorts are warranted to clarify predictive factors and guide appropriate postoperative follow-up strategies.
